# P-183. Barriers and Facilitators to Identifying and Treating Neonatal Sepsis in Ethiopia

**DOI:** 10.1093/ofid/ofae631.387

**Published:** 2025-01-29

**Authors:** Victoria A Avanzato, Ogorchi Ujari, Behayilu Yizengaw, Conor McCann, Hiwot Tibeje, Abebe Gobezayehu, John Cranmer, Rachel Hall-Clifford, Senay Zerihun, Amy Rule

**Affiliations:** Stanford Medicine, Mountain View, California; University of Cincinnati/Cincinnati Children's Hospital Medical Center, Atlanta, Georgia; Bahir Dar University College of Health Sciences, Bahir Dar, Amara, Ethiopia; Emory University College of Arts and Sciences Department of Anthropology, Atlanta, Georgia; Addis Ababa University, Addis Ababa, Adis Abeba, Ethiopia; Bahir Dar University College of Health Sciences, Emory University School of Nursing, Atlanta, Georgia; Emory University School of Nursing, Emory University College of Arts and Sciences Center for the Study of Human Health, Rollins School of Public Health, Atlanta, Georgia; Emory University College of Arts and Sciences Center for the Study of Human Health, Atlanta, Georgia; Bahir Dar University College of Health Sciences, Bahir Dar, Amara, Ethiopia; Emory University School of Medicine, Children's Health Care of Atlanta, Atlanta, Georgia

## Abstract

**Background:**

Neonatal sepsis is a major cause of death of children under the age of 5 years globally and in Ethiopia. The high mortality associated with neonatal sepsis is especially pronounced in low-birth weight infants (LBW). Antimicrobial resistance (AMR) further contributes to high mortality. Many hospitalized LBW newborns with sepsis are admitted to NICUs but mortality remains high, partly due to increasing AMR but also due to delayed identification and treatment. Thus, evaluating and describing the existing barriers to sepsis diagnosis and management is urgently needed. This may guide targeted innovations to predict, diagnose, and treat neonatal sepsis earlier and avert newborn deaths.

**Methods:**

Utilizing structured chart review and focus groups we conducted a convergent mixed methods study evaluating facilitators and barriers to early identification and care of neonates admitted with sepsis to the neonatal intensive care unit at Tibebe Ghion Specialized Hospital in Bahir Dar, Ethiopia.

**Results:**

6 resident physicians and 6 nurses participated in 2 focus groups, and 142 charts were reviewed. Chart review revealed that infants with documented warning signs (such as fast breathing or failure to suck) begin an empiric course of ampicillin and gentamycin. Further diagnostic workup is often limited, and blood cultures are rarely performed. Qualitative interviews reenforced and expanded these results. Key barriers identified included limited physical resources (monitoring equipment, laboratory capacity, and imaging) and personnel barriers (limited staffing, low morale, and frequent staff transfer). Primary facilitators included understaffing, retention of experienced staff, and access to necessary equipment and diagnostics.

**Conclusion:**

Even in a higher-level hospital in Ethiopia, neonatal sepsis is challenging to identify due in part to limited diagnostic capacity and understaffing. The absence of cultures and sensitivities and other diagnostics present barriers to early identification and targeted treatment. Future studies could focus on interventions to expand lab capacity and diagnostics, including point of care diagnostics for sepsis or AMR, capacity building of neonatal nursing, and expansion of access to low-cost vital sign monitoring.

**Disclosures:**

**All Authors**: No reported disclosures

